# Optimization and Encapsulation of Phenolic Compounds Extracted from Maize Waste by Freeze-Drying, Spray-Drying, and Microwave-Drying Using Maltodextrin

**DOI:** 10.3390/foods10061396

**Published:** 2021-06-16

**Authors:** Hojjat Pashazadeh, Oscar Zannou, Mohamed Ghellam, Ilkay Koca, Charis M. Galanakis, Turki M. S. Aldawoud

**Affiliations:** 1 Food Engineering Department, Faculty of Engineering, Ondokuz Mayis University, 55139 Samsun, Turkey; zannouoscar@gmail.com (O.Z.); mohamed.gh2010@gmail.com (M.G.); itosun@omu.edu.tr (I.K.); 2 Research & Innovation Department, Galanakis Laboratories, 73100 Chania, Greece; 3 Food Waste Recovery Group, ISEKI Food Association, 1190 Vienna, Austria; 4 Department of Botany & Microbiology, College of Science, King Saud University, Riyadh 11451, Saudi Arabia; tdawoud@ksu.edu.sa

**Keywords:** antioxidant activity, phenolic compounds, cornsilk, encapsulation techniques

## Abstract

Cornsilk is maize waste containing phenolic compounds. In this study, freeze-drying, spray-drying, and microwave-drying techniques were evaluated for the encapsulation of cornsilk’s phenolic compounds using maltodextrin as wall material. The results of antioxidant properties showed that freeze-drying was more efficient than microwave-drying and spray-drying techniques. The highest recovery of phenolic compounds was obtained with freeze-drying. The microstructure, DSC, and FTIR data showed that the encapsulation process was effective, and freeze-drying was the best drying technique. The physical properties of the microparticles greatly changed with the drying techniques. This study revealed that the phenolic compounds of the cornsilk extract can be successfully encapsulated and valorized.

## 1. Introduction 

Nowadays, the world faces challenging crises related to health, demography, and nutrition, which require innovative and sustainable solutions. New sources of food and bioactive compounds are being investigated as adequate solutions for health and nutrition. Food bioactives were reported to reinforce the immune system against COVID-19 disease and promote human health [1]. They can help prevent many diseases, including cancers, coughing, inflammatory, cardiovascular, and oxidative stress diseases [2]. The phenolic compounds are one of the most popular bioactive compounds that have a wide range of biological functions and are used for food formulations and pharmaceutical industries [3,4]. The phenolic compounds are widely found in plants, fruits, and wastes. While seeking health and nutrition solutions, the valorization of food wastes for the recovery of phenolic compounds became imperative. 

Although bioactives, mainly phenolic compounds, exhibited numerous beneficial effects, they are chemically unstable in neutral pH environments and when exposed to oxygen [5,6]. Furthermore, the chemical structure of phenolic compounds counts unsaturated bonds, which may change the structure and functions of the original compound when exposing to light, oxygen, heat, enzymatic activity, and water [7,8,9]. Thus, encapsulation techniques were developed to protect the phenolic compounds and improve their bioavailability. Moreover, the encapsulation process can also prevent unwanted taste and odor and extend the shelf-life and usages of phenolic compounds [10,11]. The encapsulation process consists of rendering the bioactive compounds in a solution of wall material, followed by drying [12]. The efficiency of encapsulation depends on the coating material and drying techniques [13,14] and the solution homogeneity. Additionally, the optimum concentration of the active compounds, the release mechanism, the final particle size, density, stability requirements, and cost constraints are other essential features to be taken into account [3]. Many coating materials are used for the microencapsulation, including maltodextrin, gum arabic, pectin, proteins, lipids, cyclodextrins, chitosan, and xanthan their combination. These materials’ efficacy depends on the preparation parameters of emulsion and features of the targeted compounds [9,14]. 

Cornsilk is the byproduct of maize (*Zea maize* L.), which is discarded after taking the corn. In ancient times, cornsilk was used in traditional medicine to treat kidney problems [15]. It was also reported to reduce hyperglycemia by incrementing insulin and recovering the beta-cells and demonstrated diuretic and kaliuretic properties at specific doses [15,16]. It has shown antioxidant activity [15,17], antimicrobial, and antidepressant properties [15,16]. Cornsilk has been used to treat cystitis, edema, gout, rheumatoid, and arthritis [16,18]. Cornsilk has proven beneficial effects in reducing obesity [19,20] and protecting against radiation-induced oxidative stress [21]. In addition, the cornsilk extract is anticancer since it induced apoptosis via the ROS-mediated mitochondrial pathway in MCF-7 cells [22]. These biological activities rely on bioactive compounds such as alkaloids, steroids, flavonoids, phenolic acids, and terpenoids that cornsilk contained [16,23,24,25]. 

Corn is the third most-produced cereal crop globally after wheat and rice [26] and consumed boiled and grilled or used to make flour, starch, oil, and syrup. The processing of corn results in a high amount of cornsilk, which is not adequately valorized. It would be beneficial to find a novel approach in the valorization of cornsilk. The phenolic compounds of cornsilk could be extracted to produce natural food additives using well-studied conventional and emerging technologies [27,28,29]. 

The recovered phenolic compounds could find different applications in food, cosmetics, and pharmaceutical industries. However, the investigation of their encapsulation and product formation is also critical to ensure their functionality [30]. To this end, the present study considers the optimization of phenolic compound extraction from cornsilk and its encapsulation with maltodextrin using freeze-drying, spray-drying, and microwave-drying.

## 2. Material and Methods

### 2.1. Plant Material

**Figure 1:**
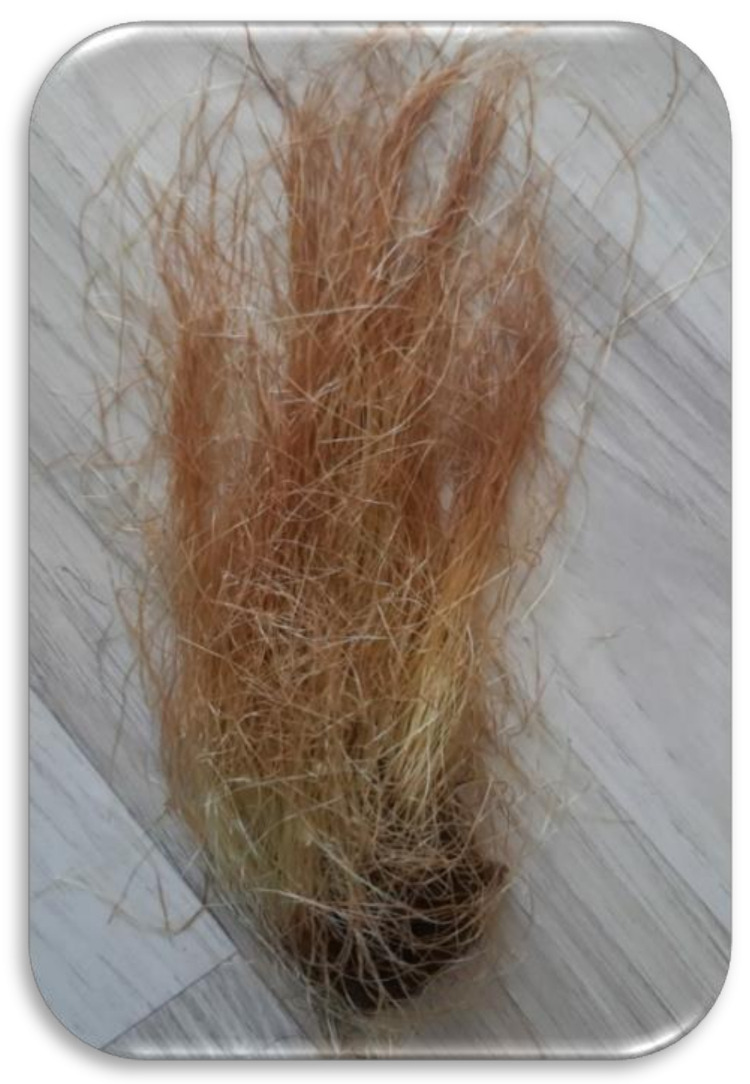
Cornsilk collected from maize (*Zea mays* Intendata).

The maize was collected from a field in Samsun/Turkey during the October 2020 season. The cornsilk was separated, washed and stored at −20 °C (Figure 1).

### 2.2. Chemical and Reagents

2,4,6-Tris(2-pyridyl)-1,3,5-triazine (TPTZ), 2,2-Diphenyl-1-Picrylhydrazyl (DPPH), 6-hydroxy 2,5,7,8-tetramethylchroman-2-carboxylic acid (Trolox), hydrochloric acid (HCl, 37%), methanol (99,8%), acetone, sodium nitrite, sodium hydroxide Folin–Ciocalteau reagent, maltodextrin and standards were purchased from Sigma-Aldrich (St. Louis, MO, USA). Gallic acid and sodium carbonate from taken from Riedel-de Haen. Sodium acetate and glacial acetic acid were taken from Carlo Erba. Aluminum chloride and iron chloride were purchased from Merck.

### 2.3. Obtaining Cornsilk Extract

A predefined amount of cornsilk was mixed with 100 mL of distilled water. The mixtures were extracted in a water bath (Nuve, ST 30) at different times and temperatures. Afterward, the extracts were filtered and diluted adequately for further analyses.

### 2.4. Optimization Design

The extraction parameters such as solid load (amount of sample in g), time (min), and temperature (°C) were optimized to provide the highest yield of total phenolic content (TPC), total flavonoid content (TFC), ferric reducing antioxidant power (FRAP), and DPPH radical scavenging activity (DPPH). The optimization was carried out using the three-level central composite design (Design expert software 11.0). The solid load, time, and temperature were assigned to the coded variables of X_1_, X_2,_ and X_3_, respectively (Table 1). The combinations of the solid load (0.13, 0.5, 1, 1.5 and 1.87 g), time (8.04, 30, 60, 90 and 111.96 min), and temperature (25.36, 40, 60, 80, and 94.64 °C) generated 17 experimental points, counting three replicates at the central point. TPC, TFC, FRAP, and DPPH radical scavenging activity values were the responses (*Y*) (Table 2). The experimental data fitted to the polynomial model in Equation (1):

(1) $$Y=\beta_{0}+\overset{3}{\underset{n=1}{\sum }}\beta_{i}X_{i}+\overset{3}{\underset{i=-}{\sum }}\beta_{ii}X_{ii}+\overset{2}{\underset{i=1}{\sum }}\overset{3}{\underset{j=i+1}{\sum }}\beta_{ij}X_{i}X_{j}$$

where *X_i_* and *X_j_* are the coded independent variables, *β_ij_*, *β_ii_*, and *β_i_* interactive, quadratic, and linear coefficients, respectively, *β* is the model intercept, and *Y* is the predicted response.

### 2.5. Total Phenolic Content (TPC)

The TPC was determined using Folin–Ciocalteu method as described in Zannou and Koca [31]. The absorbance was read at 760 nm using a UV- spectrometer (Thermo Spectronic) and compared to a standard curve as gallic acid equivalent (mg GAE/g).

### 2.6. Total Flavonoid Content (TFC)

The TFC was determined using the protocol [31]. The absorbance was read at 510 nm, and TFC was calculated following the calibration curve of epigallocatechin. The results were given as mg epigallocatechin equivalents (ECE) per g.

### 2.7. DPPH Radical Scavenging Activity Assay

The DPPH assay was carried out following the method of Zannou et al. [32]. The mixture was placed in the dark for 1 h at room temperature, followed by the absorbance recording at 517 nm. The DPPH solution was used as a control, and the scavenging ratio was calculated with Equation (2).

(2) $$\mathrm{Reduction}\;\left(\%\right)=\left(\frac{A_{c}-A_{s}}{A_{c}}\right)\times 100$$

where *A_c_* is the absorbance of the control and *A_s_* is the absorbance of extract.

### 2.8. Ferric Reducing Antioxidant Power (FRAP) Assay

FRAP assay performed according to the procedure of Zannou et al. [32] The FRAP values of the extracts were calculated from a calibration curve using FeSO4 as standard. The results were given as mmol FeSO4 equivalents (mmol ISE/g).

### 2.9. Encapsulation Process

For the encapsulation of the phenolic compounds of maize byproduct aqueous extracts, three techniques (freeze-drying, spray-drying, and microwave-drying) were used and compared. Maltodextrin (4.0–7.0 DE) was used as a coating agent. Maltodextrin and extract mixture was prepared by mixed 10 g of maltodextrin with 90 g of cornsilk aqueous extract. The mixture was heated at 60 °C for 1h and then subjected to ultraturax at 11,000 rpm for 5 min. The spray drying was performed using a Buchi mini spray dryer B-290 (Noble Park, VIC, Australia) as described in [33]. The freeze-drying was carried out by initially frozen the samples using liquid nitrogen and then freeze-dried for 48 h (FD3 freeze dryer, Thomas Australia Pty. Ltd., Seven Hills, NSW, Australia) [33]. The microwave drying was performed in a domestic microwave oven (Vestel, Turkey) at microwave power intensity of 750 W for 10 min, selected based on the preliminary experiments.

### 2.10. Production Yield

The production yield (*PY*) is the percentage of the concentration ratio in powder to the total concentration in the extract. *PY* of TPC, TFC, DPPH radical scavenging and FRAP was determined with Equation (3).

(3) $$PY=\frac{\mathrm{Concentration}\;\mathrm{in}\;\mathrm{powder}}{\mathrm{Total}\;\mathrm{concentration}\;\mathrm{in}\;\mathrm{the}\;\mathrm{extract}}\times \;100$$

### 2.11. Surface Phenolic Content and Efficiency of Phenolic Compounds

The surface phenolic content was determined by the method of Cilek et al. [14]. The encapsulation efficiency of microcapsules’ phenolic content was calculated according to Equation (4).

(4) $$EE=\frac{\mathrm{Total}\;\mathrm{phenolic}\;\mathrm{content}-\mathrm{Surface}\;\mathrm{phenolic}\;\mathrm{content}}{\mathrm{Total}\;\mathrm{phenolic}\;\mathrm{content}}\times \;100$$

### 2.12. Physico-Chemical Characteristics

The moisture content was measured with an oven at 70 °C for 24 h. The solubility was determined according to the method used by Fazaeli et al. [34] The bulk density and tapped density were determined according to [35]. Carr index was calculated from the bulk and tapped densities of the powders as expressed in Equation (5).

(5) $$Carr\;index=\frac{Tapped\;density-Bulk\;density}{Tapped\;density}$$

### 2.13. Microstructure Analysis

The microstructure analysis was performed using a scanning electron microscope (SEM, JEOL JSM-7001F). A small specimen was briefly taken from the samples and attached to a stainless stub with double sticky tape. The assembly was then immediately sputtered with a gold/palladium target (60/40) in approximately 10 nm using a sputter coater, functioning with argon and plasma current for 2 min. The images were recorded at an acceleration voltage of 10 kV and 15 kV.

### 2.14. Differential Scanning Calorimetry (DSC) Analysis

The differential scanning calorimeter (New Castle, DE; TA Instrument 2010) was used for the calorimetric measurements and calibrated with indium. The calorimetric scans’ temperature range varied from 10 to 60 °C, under nitrogen flow. The scan rate was set to 5 °C min^−1^. The Tm and ∆H were determined from the thermograms by the Universal 4.0 C. Software.

### 2.15. Fourier Transformed Infrared (FTIR) Analysis

FTIR analysis was carried out according to [32] using an FTIR spectrometer (Perkin Elmer, Spectrum-Two, USA, PEService 35).

### 2.16. LC/MS/MS Analysis

The phenolic compounds were determined using liquid chromatography coupled to a mass spectrometer detector (LC-MS/MS, Shimadzu LC-MS 8040) via electrospray ionization (ESI) and two pumps (LC-30 AD), a column oven (CTO-10AS VP), an autosampler (SIL-30AC) and a degassing unit (DGU-20A 3R), according to Zannou et al. [36] The phenolic compounds were identified based on their elution time and quantified from their peak area. The identified compounds were quantified using a mixture of external standards (protocatechuic aldehyde, protocatechuic acid, gallic acid, catechin, vanilin, ρ-cumaric acid, caffeic acid, ferulic acid, 4-hydroxy benzoic acid, salicylic acid, ellagic acid and quercetin) at different concentrations.

### 2.17. Data Analysis

The Design-Expert software 9.0 (Trial version, Stat-Ease Inc., Minneapolis, MN, USA) was used to design the optimization process and to generate the models and 3D graphics. The ANOVA of response surface methodology was applied for the statistical significance. The coefficient of determination (R^2^), adjusted coefficient of determination (adj. R^2^), coefficient of variation (CV), and Fisher’s test value (F-value) were used for the validation of the models. The models and terms were considered significant at *p* < 0.05. The optimum conditions were determined based on the desirability function. Other analyses were carried out in three replicate, the one-way ANOVA with posthoc Duncan’s test was applied (SPSS, version 21) and the results were considered at *p* ≤ 0.05.

## 3. Results and Discussion

### 3.1. RSM Results

#### 3.1.1. Model Analysis

The optimization process was performed on the extraction conditions of solid load (amount of sample in g), time (min), and temperature (°C) to have the highest yield of TPC, TFC, FRAP, and DPPH radical scavenging activity. The coded and responses results of the experimental runs were shown in Table 2. The responses were ranges of 20.30–50.82 mg GAE/g, 2.19–7.20 mg ECE/g, 31.83–112.83 mmol TE/g, and 58.39–178.06 mmol ISE/g for TPC, TFC, DPPH radical scavenging activity, and FRAP values, respectively. Nurhanan and Wan Rosli [17] have reported ranges of 6.70–101.99 mg GAE/g for TPC and 0.66–9.26 mg catechin equivalent/g for TFC in cornsilk extracted with water, methanol, ethanol, and ethyl acetate. Ebrahimzadeh et al. [37] have reported 118.94 mg GAE/g and 58.22 mg quercetin equivalent/g for TPC and TFC, respectively. Our results were higher than those reported previously. The highest findings detected in the cornsilk studied in this study might be related to corn species, production, and extraction analysis conditions. During the RSM process, the highest TPC was found at run 14 (0.5 g, 90 min, and 80 °C) and the highest TFC at run 16 (0.5 g, 30 min, and 80 °C). The most increased DPPH radical scavenging activity and FRAP were found at run 16 (0.5 g, 30 min, and 80 °C) and run 11 (0.13 g, 60 min, and 60 °C). It was observed that the dependent variables responded differently with each combination of independent variables. Such observation was observed previously during the optimization for the extraction conditions [31,38]. The significance of regression coefficients of polynomial models was set at *p* < 0.05. The reduced second-order models of coded units were stepwise determined and expressed as:

(6) $$TPC=28.94-2.97X_{1}+3.00X_{2}+8.68X_{3}-1.26X_{1}X_{2}-1.55X_{1}X_{3}\;-0.2131X_{2}X_{3}+2.37X_{1}^{2}-0.1785+1.59X_{3}^{2}$$

(7) $$TFC=4.57-0.35X_{1}+0.13X_{2}+1.42X_{3}+0.19X_{1}X_{2}-0.17X_{1}X_{3}-0.48X_{2}X_{3}\;-0.15X_{1}^{2}-0.04X_{2}^{2}-0.04X_{3}^{2}$$

(8) $$DPPH\;radical\;scavenging=71.70-11.47X_{1}+4.17X_{2}+17.79X_{3}+0.45X_{1}X_{2}\;-11.82X_{1}X_{3}-3.45X_{2}X_{3}-0.30X_{1}^{2}-3.37X_{2}^{2}-3.20X_{3}^{2}$$

(9) $$FRAP=99.68-21.03X_{1}+4.17X_{2}+25.58X_{3}+0.50X_{1}X_{2}-8.12X_{2}X_{3}\;-4.18X_{2}X_{3}+8.27X_{1}^{2}-5.03X_{2}^{2}-1.91X_{3}^{2}$$

The results of ANOVA of the responses given by RSM were assembled in Table 3. The model TPC is significant (*p* < 0.0185) and presented relatively higher R^2^ (0.8741), adjusted R^2^ (0.7121), and adequate precision (7.5136). This adequate precision referred to the desired signal-to-noise ratio, which means that the model can be used to navigate the design space. The lack of fit (F-value 1.19) is insignificant due to the pure error, indicating that the model is well fitted. For the model generated for TFC, the model is significant (*p* < 0.0025) and characterized by higher R^2^ (0.9324), adjusted R^2^ (0.8455), predicted R^2^ (0.5045), and adequate precision (11.1069). Its lack of fit is non-significant with F-value 6.11 (*p* < 0.1466). Thus, the model had a good fit.

The model of DPPH radical scavenging is significant (*p* < 0.0005), exhibiting higher R^2^ (0.9583), adjusted R^2^ (0.9048), predicted R^2^ (0.7158), and adequate precision (13.8924). The predicted R^2^ is in reasonable agreement with the Adjusted R^2^, and the sufficient accuracy of 13.892 indicates that the model can be used to navigate the design space. The lack of fit (F-value 1.85) is not significant, implying that the model is a good fit. The model developed for FRAP is significant with F-value (*p* < 0.0052) and presented greater R^2^ (0.9152) and adjusted R^2^ (0.8061). The adequate precision of 9.3927 is desirable, and the model is sufficient for the navigation of the design space. The lack of fit is insignificant (F-value 1.71), suggesting that the model had a desirable fit.

#### 3.1.2. RSM-Effects of Independent Variables on the Responses

The effects of different terms of independent variables on the responses were given in Table 3. The linear terms of solid load and extraction time had no significant impact on TPC and TFC, respectively, while the linear terms of temperature displayed a significant effect. Likewise, the linear terms of solid ratio and temperature presented significant DPPH radical scavenging and FRAP results. No quadratic and interaction units showed substantial impacts on the responses. Previously, it was demonstrated that the RSM linear and quadratic terms react differently depending on the samples’ intrinsic characteristics, many independent variables, and analysis conditions [32,38].

**Figure 2:**
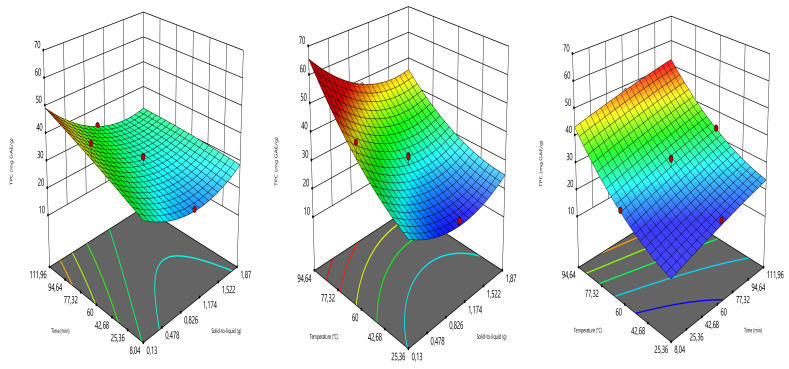
Three-dimensional graphics showing the effects of the independent variables on TPC.

**Figure 3:**
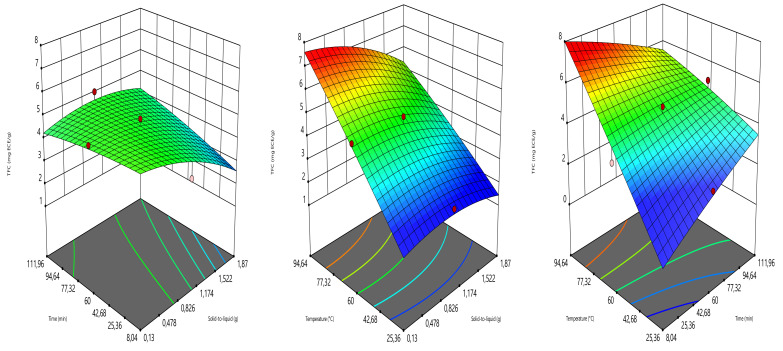
Three-dimensional graphics showing the effects of the independent variables on TFC.

**Figure 4:**
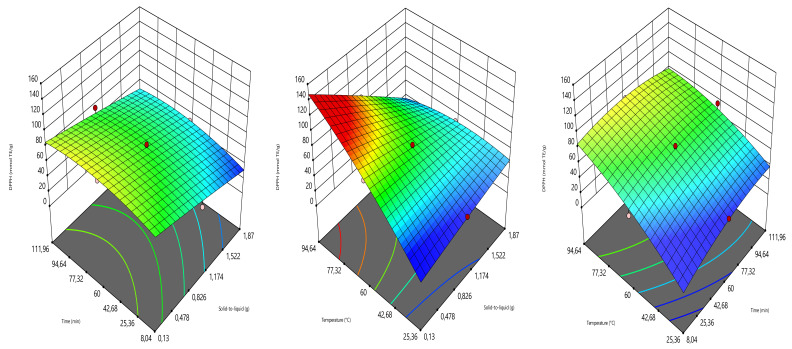
Three-dimensional graphics showing the effects of the independent variables on DPPH.

**Figure 5:**
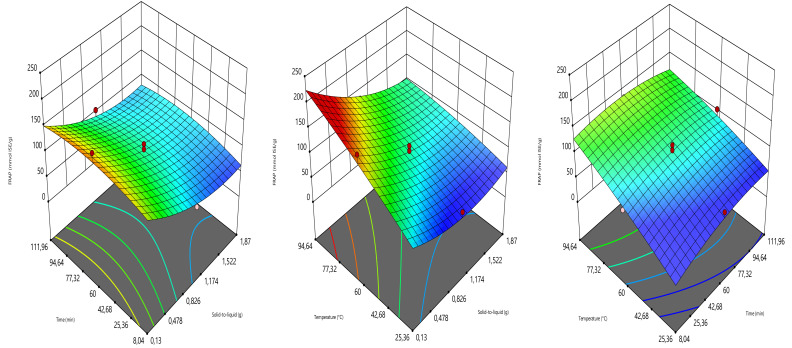
Three-dimensional graphics showing the effects of the independent variables on FRAP.

The 3D graphics showing the impacts of combining the independent variables on the responses were given in Figure 2, Figure 3, Figure 4 and Figure 5. As can be seen, the TPC augmented greatly when increasing the extraction time and temperature. Similarly, Nurraihana et al. [39] have reported that the TPC increased when increasing the extraction time. The solid-to-liquid ratio exhibited a moderate effect on TPC, where the TPC decreased from 0.13 to 1.50 g and then increased.

For the TFC, the different combinations of the independent variables responded diversely (Figure 3). In the combination of decoction time and solid-to-liquid ratio, the decoction time did not show a significant effect on TFC, while the solid-to-liquid ratio had a significant effect on this response. The TFC showed the highest values at 0.13–1.17 g and then decreased. In the combination of temperature and solid-to-liquid ratio, the solid-to-liquid ratio was almost indifferent while the temperature-induced a strong increase in TFC when augmenting. Whereas the increase in the combination of temperature and time exhibited a great increase in TFC.

As can be seen in Figure 4, the temperature and decoction time have mostly influenced the response DPPH. The amount of this latter increased with the increase in the temperature and decoction time, while almost with solid-to-liquid ratio. Similarly, Nurraihana et al. [39] have reported that the DPPH values increased with the increase the extraction time.

The independent variables have greatly influenced the response FRAP (Figure 5). The increase in temperature and decoction time induced the increase in FRAP. However, FRAP showed a decrease at solid-to-liquid ratio ranged from 0.13 to 1.52 g and then increased.

#### 3.1.3. Multi-RSM

The application of the desirability function determined the optimum conditions to have the maximum responses. The optimum extraction conditions at desirability 0.871 were 0.5 g, 62.10 min, and 80 °C giving 46.39 mg GAE/g, 6.29 mg ECE/g, 109.28 mmol TE/g, and 160.72 mmol ISE/g for TPC, TFC, DPPH radical scavenging, and FRAP, respectively. Further analyses were performed at the optimum conditions for confirmation and the experimental results were found close to the predicted values as 58.87 ± 1.38 mg GAE/g, 6.64 ± 0.12 mg ECE/g, 132.63 ± 1.66 mmol TE/g, and 172.30 ± 3.40 for TPC, TFC, DPPH radical scavenging, and FRAP, respectively. The encapsulation process was performed at the optimum experimentation conditions.

### 3.2. Phytochemical and Antioxidant Features of Extract and Powders

Under these conditions, the experimental results were 58.87 ± 1.38 mg GAE/g, 6.64 ± 0.12 mg ECE/g, 132.63 ± 1.66 mmol TE/g, and 172.30 ± 3.40 for TPC, TFC, DPPH radical scavenging, and FRAP, respectively. In contrast, previous studies have reported fewer antioxidant features that can be associated with corn cultivars, cultivation, and analysis conditions [17,37,39]. Nine phenolic compounds were identified from cornsilk, and protocatechuic acid (205.21 µg/g) was the most abundant, followed by ρ-coumaric acid (42.67 µg/g), caffeic acid (36.87 µg/g), and ferulic acid (12.11 µg/g) (Table 4). Similarly, Aires and Carvalho [40] have found out that the main phenolic compounds in cornsilk collected from Portugal were ferulic acid (45.2 µg/g), followed by chlorogenic acid (39.9 µg/g), caffeic acid (12.9 µg/g), apigenin (7.4 µg/g) and pelargonidin (2.4 µg/g).

As can be seen in Table 4, the freeze-drying was the most efficient drying method compared to other drying methods, providing the highest values of protocateuic acid (152.06 µg/g), ρ-cumaric acid (112.83 µg/g), vanilin (17.79 µg/g), salicylic acid (17.32 µg/g) and catechin (5.58 µg/g). These findings were in agreement with Ballesteros et al. [41] who have found out that maltodextrin was the best wall material and freeze-drying the best drying technique for the encapsulation of antioxidant phenolic compounds. Gallic acid and ferulic acid disappeared during the drying processes, while ellagic acid appeared in spray and microwave-dried samples. Catechin and ellagic acid were probably under the detection limit for the extract but they were found in certain powders. Thus, the drying processes differently affected the individual phenolic compounds. Protocatechuic aldehyde, gallic acid, vanillin, salicylic acid, and 4-hydroxybenzoic acid were also identified in the present study as minor phenolic compounds. The extract obtained after the application of RSM displayed a high amount of protocatechuic acid and the freeze-drying protected these compounds better. Protocatechuic acid has great antioxidant activity and salicylic acid is well-known to have analgesic, antipyretic anti-inflammatory properties.

### 3.3. Encapsulation Efficiency

The efficiency of the drying processes (freeze-drying, spray-drying, and microwave-drying) after the encapsulation was evaluated and showed in Table 5. The best results were achieved using the freeze-drying technique. Under these conditions, the percentage of TPC, TFC, DPPH radical scavenging, and FRAP retained in the encapsulated samples were 98.85 ± 0.90%, 97.95 ± 4.21%, 70.39 ± 6.20%, 82.45 ± 0.89%, respectively. These results follow those reported previously, where the highest amount of retention of antioxidant activity and phenolic compounds in the encapsulated samples was obtained with freeze-drying and 100% maltodextrin [41]. The freeze-drying was followed by spray-drying and microwave-drying processes. Regarding the efficiency of encapsulation, the drying technique was revealed to be the fundamental factor. The efficiency given by the freeze-drying method could be partially attributed to the morphological changes occurring in the drying process. The freeze-drying resulted in the sawdust-like shape, which creates a lower surface area/volume ratio. Simultaneously, the spray-drying generates a larger surface area, allowing the phenolic compounds of the surface to alter [41].

The surface phenolic contents were low which denoted higher efficiency of the encapsulation of phenolic compounds (Table 5). As can be seen in Table 4, the freeze-drying was the most efficient drying method compared to other drying methods, providing the highest values of protocatechuic acid (152.06 µg/g), ρ-cumaric acid (112.83 µg/g), vanillin (17.79 µg/g), salicylic acid (17.32 µg/g) and catechin (5.58 µg/g). These findings agreed with Ballesteros et al. [41], who have found out that maltodextrin was the best wall material and freeze-drying the best drying technique for the encapsulation of phenolic compounds. Gallic acid and ferulic acid disappeared during the drying processes, while ellagic acid appeared in spray and microwave-dried samples. Thus, the drying processes affected the individual phenolic compounds differently.

### 3.4. Scanning Electron Microscopy

**Figure 6:**
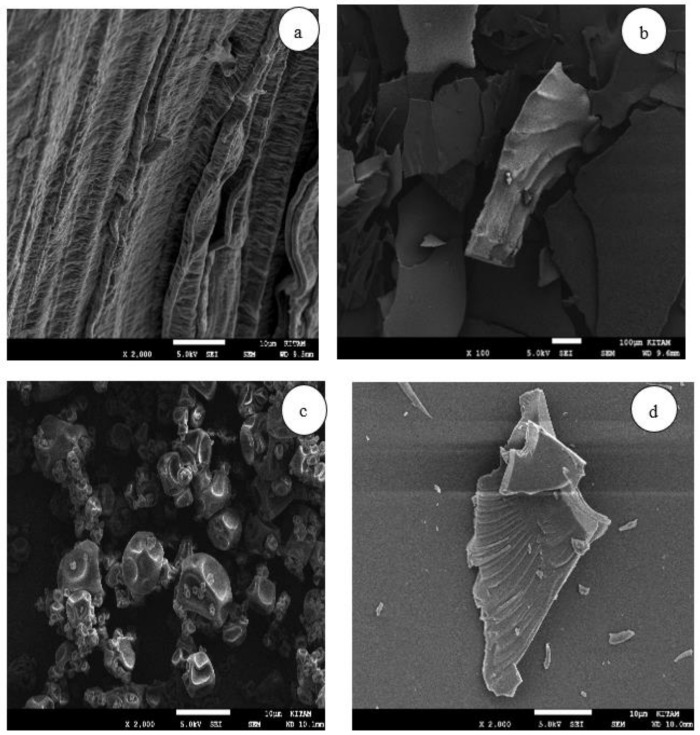
Microstructures of waste extract (**a**), freeze-dried (**b**), spray-dried (**c**), and microwave-dried (**d**) samples.

The micrography of the extract showed amorphous and homogenous features (Figure 6a). These features were reported to be the non-purification of the extract [9]. The introduction of maltodextrin together with the drying processes affected the shape and size of the microcapsules differently. The microcapsules were obtained from the freeze-drying broken plate surface and sawdust-like morphology (Figure 6b). Similar morphology was reported for freeze-drying [9,41]. The microcapsules obtained from the spray-drying were irregularly spheric with heterogeneous sizes, where some small particles were inserted in the middle of bigger dimension particles (Figure 6c). This morphology was observed for many products’ spray-drying [33,42]. The image of powder issued from microwave-drying (Figure 6d) revealed an amorphous microcapsule with irregularly-shaped smaller particles suspended on its surface. The uniformism of the microstructure of freeze-drying increased the encapsulation powder, protecting more the phenolic compounds. However, the variations in morphological changes for spray-drying and microwave-drying processes might alter the encapsulation power since the variation in the surface area occurring during the encapsulation process provokes more or less degradation of the encapsulated compounds [41].

### 3.5. Differential Scanning Calorimetry (DSC)

**Figure 7:**
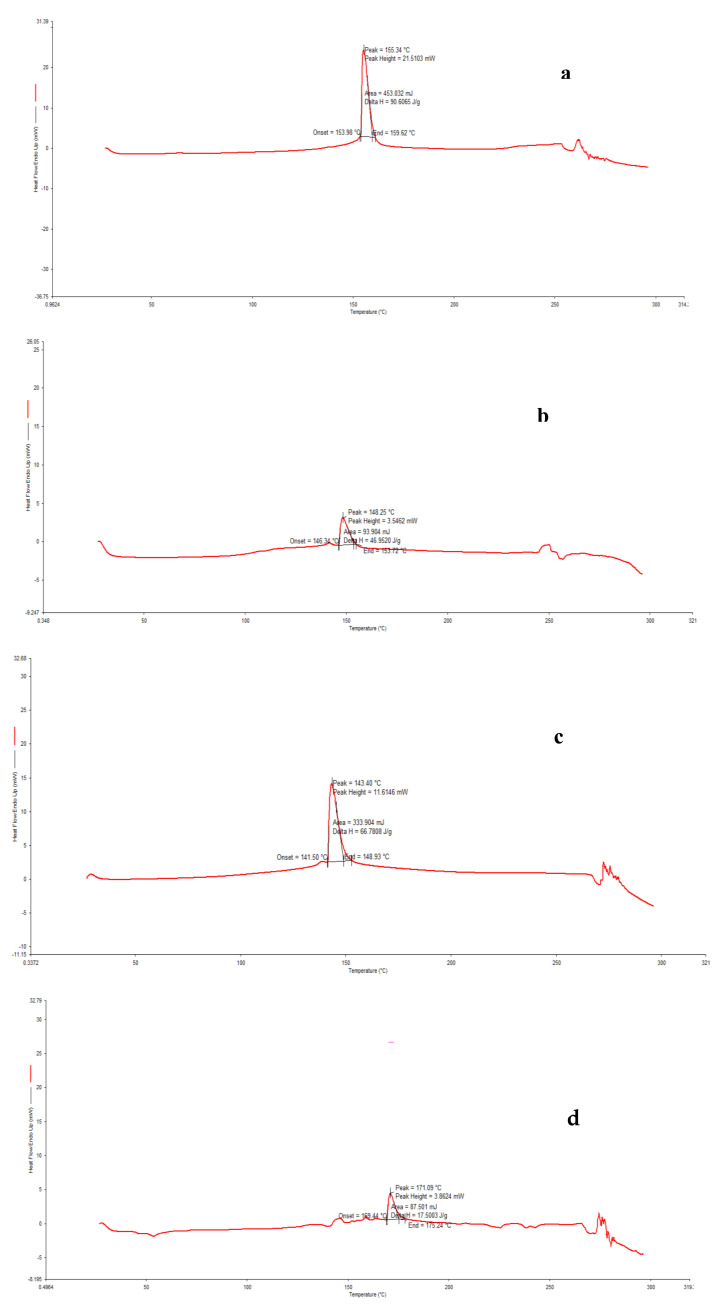
DSC thermograms of maltodextrin (**a**), freeze-dried (**b**), spray-dried (**c**), and microwave-dried (**d**) samples.

Figure 7 showed the thermograms of maltodextrin and capsules obtained from differential scanning calorimetry and the glass transition temperatures were given in Table 6. Figure 7a exhibited an endothermic peak at 155.34 °C with an onset at 153.98 °C and an endpoint at 159.62 °C, corresponding to the glass transition temperature maltodextrin (Figure 7a). Similarly, Kurozawa et al. [43] and Deschamps et al. [44] have reported a glass transition temperature of 160 °C for maltodextrin.

As can be seen, the encapsulated products’ glass transition temperatures were close to the value of the encapsulating agent, guaranteeing the effectiveness of the encapsulation processes. After freeze-drying and spray-drying, the partial disappearance of the thermal events (Figure 7b,c) was observed with lower glass transition temperatures of 148.25 °C and 143.40 °C, respectively, when compared to the glass transition temperature of the encapsulating agent. This observation denoted that the guest molecule 46es integrated the encapsulating agent to form inclusive complexes [45,46]. Regarding the microwave-drying (Figure 7d), the glass transition temperature of the encapsulated product (171.09 °C) was higher than the value of the glass transition temperature of an encapsulating agent, suggesting that the desolvation or loss of the guest molecules occurred [47].

### 3.6. Fourier Transform Infrared (FTIR) Analysis

**Figure 8:**
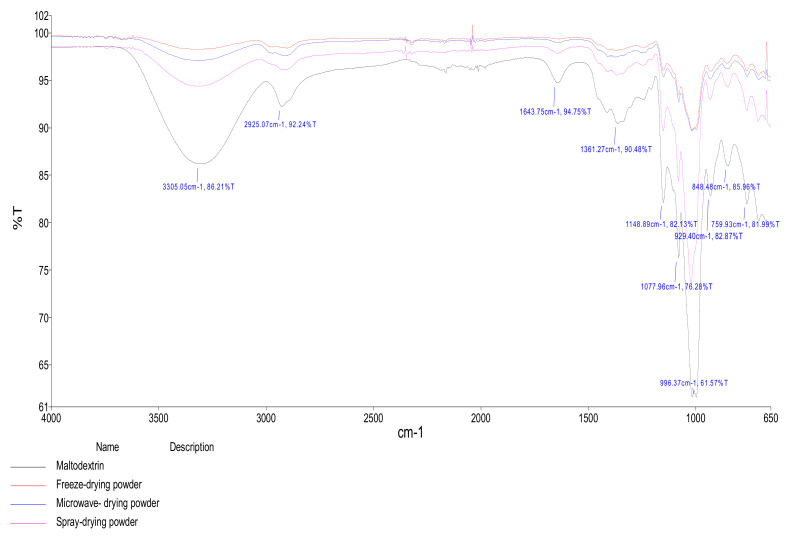
FTIR diagrams of maltodextrin and encapsulated products.

The FTIR spectra of encapsulated phenolic compounds of cornsilk in maltodextrin using freeze-drying, spray-drying, and microwave-drying showed several relevant peaks (Figure 8). The FTIR spectra were in the range of 500–4000 cm^−1^ tendencies for the wall material and freeze, microwave, and spray-dried products. In all samples, the peaks existent in frequencies between 2800 and 3700 cm^−1^ were predominantly assigned to hydrogen bonds (O-H stretch), carboxylic acids, and residual water [48,49]. The peak at 2900 cm^−1^ frequency was related to the symmetric and asymmetric stretching of the C-H bond in methyl groups [49] or belonged to the aromatic C-H bond. The same peak at 2930 cm^−1^ frequency was linked to C-H_2_, *n*C-H_3_ stretching of polyphenolic compounds [41]. The peaks at 1600–1650 cm^−1^ frequencies associated to C-C or C=C bonds, 1063 cm^−1^ to C-O or C-O-C bonds, at 1450–1300 cm^−1^ bonds to -CH2, -CH, and =CH bonds were assigned to carbohydrates of maltodextrin [49] and phenolic compounds [49,50,51]. The FTIR spectra presented similar peaks for the wall material and freeze, microwave, and spray-dried products, indicating that the encapsulating agent successfully covered the extracts. Moreover, the peaks of the spectra of freeze, microwave, and spray-dried products were more pronounced, suggesting that the extracts were incorporated in maltodextrin [52].

### 3.7. Physical Characteristics of Powders

The physical properties of encapsulated samples of cornsilk are given in Table 7. The matter was from 98.10%, 98.14% and 98.23% in microcapsules dried with freeze-drying, spray-drying and microwave-drying, respectively. The water activity (a_w_) is the availability of free water in a food system responsible for biochemical activities. The microcapsules produced by spray-drying had significantly higher water activity (0.1627 ± 0.0004) than those produced by microwave-drying (0.0873 ± 0.0245) and freeze-drying (0.0401 ± 0.0034). According to Belscak-Cvitanovic et al. [53], low water content (less than 5%) guarantees long-term stability and adequate packing as well as prolonged storage. The powders produced by spray-drying, microwave-drying, and freeze-drying could be considered microbiologically and enzymatically stable as they displayed water activities lower than 0.60 [33,54]. Our results are in agreement with those reported by Papoutsis et al. [33], who have observed that the citrus byproduct extracts encapsulated by spray-drying using soybean protein isolate or ι-carrageenan as encapsulating agents had higher water activity than those encapsulated by freeze-drying.

The microcapsules had good solubility of 90.45% in freeze-drying, 95.95% in spray-drying and 95.95% in microwave-drying samples. The highest solubility was found with spray-drying and in microwave-drying samples because produced small size particles which submerged and dissolved rapidly in water. High solubility is desired since it increases the bioavailability of the encapsulated products [55,56]. In accordance with several studies [55,57], the present study revealed high solubilities (>90%) of the powders produced with maltodextrin. Independently to the drying techniques, the use of maltodextrin as the encapsulating agent facilitated the solubility of the powders, since maltodextrin has shown good solubility attributed to its hydrophilic character [57].

The bulk and tapped bulk densities are essential criteria for storage and packaging. The powders that displayed low bulk densities are more subjected to oxidation and have low storage stability because of more air between their cavities [58]. As can be seen in Table 7, the powder produced by microwave-drying provided significantly higher bulk density (0.49 g/cm^3^) than those of spray-drying powder (0.20 g/cm^3^) and freeze-drying (0.11 g/cm^3^). Likewise, the powder produced by microwave-drying had significantly higher tapped bulk density (0.59 g/cm^3^) than those of spray-drying (0.39 g/cm^3^) and freeze-drying (0.15 g/cm^3^). Peleg and Bagley [59] have affirmed that the bulk density of food processed to powder varies from 0.4 to 0.95. Not far from our findings, Che Man et al. [60] have found that the spray-dried products had a higher density than freeze-dried products. Carr’s index is generally used to evaluate the flow properties of powders. Carr’s index was 17.00, 27.00 and 48.00% for microwave, freeze and spray-dried products, respectively. According to the classification of Turchiuli et al. [61], microwave, freeze and spray-dried samples represented good, bad and very bad flowability, respectively. The bad Carr’s index of freeze and spray-dried samples might be due to higher moisture content which causes particles to stick and increases the resistance to flow [62]. It might also due small size and irregular shape of these microcapsules [61,62].

## 4. Conclusions

The response surface methodology was applied to determine the conditions that provide the highest values of TPC, TFC, FRAP and DPPH of cornsilk. The optimum conditions were 0.5 g for solid load, 62.10 min for decoction time and 80 °C for temperature. At the optimum conditions, the predicted results were 46.39 mg GAE/g, 6.29 mg ECE/g, 109.28 mmol TE/g, and 160.72 mmol ISE/g for TPC, TFC, DPPH radical scavenging, and FRAP, respectively, whereas the experimental results were found close to the predicted values as 58.87 ± 1.38 mg GAE/g, 6.64 ± 0.12 mg ECE/g, 132.63 ± 1.66 mmol TE/g, and 172.30 ± 3.40 for TPC, TFC, DPPH radical scavenging, and FRAP, respectively. The efficiency of 98.85 ± 0.90%, 97.95 ± 4.21%, 70.39 ± 6.20%, 82.45 ± 0.89% was obtained for TPC, TFC, DPPH radical scavenging, and FRAP, respectively. The results of antioxidant properties showed that the freeze-drying provided the highest efficiency. The highest recovery of phenolic compounds, microstructure, thermograms, and FTIR of the encapsulated samples showed that the encapsulation process was successful, and the freeze-drying was the best drying technique. The glass transition temperatures were 148.25 °C, 143.40 °C, and 171.09 °C for freeze-dried, spray-dried, and microwave-dried samples. The microwave-dried samples displayed the highest bulk and tapped bulk densities, which are desirable. However, the spray-dried samples had increased water activity, while the lowest values were found in freeze-dried and microwave-dried samples. This work confirmed the feasibility of the encapsulation of phenolic compounds of a cornsilk extract with maltodextrin using freeze-drying, spray-drying, and microwave-drying. Further studies should be carried out on the bioavailability and anti-microbial properties of the encapsulated products.

## Figures and Tables

**Table 1:** Coded and actual values of independent variables.

Coded Values	Actual Values		
	Solid Load (X_1_)	Time (X_2_)	Temperature (X_3_)
−1.73	0.13	8.04	25.36
−1	0.5	30	40
0	1	60	60
1	1.5	90	80
1.73	1.87	111.73	94.64

**Table 2:** Actual units, coded units and responses of central composite design.

Run	Coded Values			Responses			
	X_1_	X_2_	X_3_	TPC, mg GAE/g	TFC, mg ECE/g	DPPH, mmol TE/g	FRAP, mmol ISE/g
1	0	0	−1, 73	20.31 ± 1.52	2.19 ± 0.012	31.83 ± 3.70	60.44 ± 0.84
2	−1	1	−1	24.27 ± 1.17	3.09 ± 0.33	52.81 ± 1.85	80.14 ± 1.52
3	−1	−1	−1	20.61 ± 0.31	2.78 ± 0.06	40.91 ± 4.84	72.94 ± 2.20
4	1	1	−1	28.71 ± 1.66	3.44 ± 0.04	49.73 ± 1.33	68.00 ± 1.35
5	1	−1	−1	19.16 ± 1.01	2.27 ± 0.042	45.02 ± 4.08	58.39 ± 0.79
6	0	1.73	0	33.84 ± 1.51	5.03 ± 0.33	75.80 ± 4.27	104.17 ± 6.79
7	0	0	0	23.05 ± 1.38	4.39 ± 0.06	75.20 ± 1.14	101.29 ± 2.72
8	0	0	0	32.08 ± 0.69	4.44 ± 0.01	65.32 ± 1.56	86.39 ± 1.53
9	0	−1.73	0	23.84 ± 0.41	3.51 ± 0.55	45.35 ± 0.71	70.53 ± 3.57
10	0	0	0	31.69 ± 0.27	4.88 ± 0.08	74.59 ± 2.28	111.38 ± 6.13
11	−1.73	0	0	46.65 ± 4.79	4.89 ± 0.06	89.45 ± 1.59	178.06 ± 5.14
12	1.73	0	0	26.34 ± 3.03	3.01 ± 0.47	50.14 ± 1.98	76.42 ± 2.18
13	1	1	1	38.13 ± 0.18	5.27 ± 0.07	60.62 ± 0.57	101.32 ± 1.47
14	−1	1	1	50.82 ± 3.49	5.69 ± 0.32	101.99 ± 2.56	146.35 ± 1.36
15	1	−1	1	40.35 ± 1.29	6.09 ± 0.20	60.76 ± 1.52	108.84 ± 0.00
16	−1	−1	1	37.08 ± 3.60	7.20 ± 0.24	112.88 ± 3.13	155.48 ± 6.13
17	0	0	1.73	47.97 ± 5.13	6.35 ± 0.78	90.33 ± 0.57	133.00 ± 7.02

X_1_ = Solid load; X_2_ = Time; X_3_ = Temperature; TPC = Total Phenolic Content; TFC = Total flavonoid content; DPPH = DPPH Radical Scavenging Activity; FRAP = Ferric Reducing Antioxidant Power.

**Table 3:** Results of ANOVA of the reduced models.

	TPC			TFC			DPPH			FRAP		
	SS	F-Value	*p*-Value	SS	F-Value	*p*-Value	SS	F-Value	*p*-Value	SS	F-value	*p*-Value
Model	1434.39	5.40	0.0185	32.81	10.73	0.0025	7954.31	17.89	0.0005	18,013.62	8.39	0.0052
X_1_	123.65	-	-	1.74	-	-	1841.40	37.27	0.0005	6190.97	25.95	0.0014
X_2_	126.37	-	-	0.23	-	-	243.05	-	-	243.74	-	-
X_3_	1055.32	35.74	0.0006	28.21	83.03	<0.0001	4431.78	89.69	<0.0001	9164.05	38.42	0.0004
X_1_X_2_	12.69	-	-	0.30	-	-	1.59	-	-	2.00	-	-
X_1_X_3_	19.25	-	-	0.24	-	-	1116.95	22.61	0.0021	528.01	-	-
X_2_X_3_	0.3632	-	-	1.83	-	-	95.45	-	-	140.06	-	-
X_1_X_1_	68.60	-	-	0.27	-	-	1.09	-	-	832.95	-	-
X_2_X_2_	0.3887	-	-	0.02	-	-	138.57	-	-	308.76	-	-
X_3_X_3_	30.73	-	-	0.02	-	-	125.09	-	-	44.43	-	-
Residual	206.67			2.38			345.87			1669.69		
Lack of Fit	154.61	1.19	0.5159	2.23	6.11	0.1466	284.50	1.85	0.3864	1353.72	1.71	0.4081
Pure error	52.06			0.15			61.38			315.97		
Total	1641.06			35.18			8300.18			19,683.31		
												
R^2^	0.8741			0.9324						0.9152		
Adj R^2^	0.7121			0.8455						0.8061		
Pred R^2^	0.1696			0.5045						0.4370		
Adeq Precision	7.5136			11.1069						9.3927		
C.V. %	16.95			13.29						15.33		

- = Insignificant terms at *p* < 0.05; X_1_ = Solid load; X_2_ = Time; X_3_ = Temperature; TPC = Total Phenolic Content; TFC = Total flavonoid content; DPPH = DPPH Radical Scavenging Activity; FRAP = Ferric Reducing Antioxidant Power.

**Table 4:** LC/MS/MS quantification of phenolic compounds identified in extract and powders.

RT	Phenolic Compounds	Concentrations µg/g			
		Extract	Freeze-Drying	Microwave-Drying	Spray-Drying
10.05	Gallic acid	1.42	n.d.	n.d.	n.d.
12.22	Protocateuic acid	205.21	152.06	n.d.	16.53
13.16	Protocatechuic aldehyde	1.82	n.d.	n.d.	21.51
13.33	Catechin	0.00	5.58	2.38	3.60
15.18	Caffeic acid	36.87	n.d.	n.d.	30.19
15.87	Vanilin	1.35	17.79	n.d.	17.78
16.91	ρ-cumaric acid	42.66	112.83	n.d.	101.30
17.18	Ferulic acid	12.11	n.d.	n.d.	n.d.
18.03	4-Hydroxy benzoic acid	1.06	6.00	5.77	23.89
18.13	Salicylic acid	1.25	17.32	3.61	10.32
19.38	Ellagic acid	n.d.	n.d.	13.98	7.41

RT is the retention time; n.d. = not identified.

**Table 5:** Productivity yield and efficiency of microencapsulated powders.

Treatments	Productivity Yield (%)					
	TPC	TFC	DPPH	FRAP	Surface Phenolic Content (mg GAE/g)	Efficiency (%)
Microwave-drying	82.29 ± 0.45c	71.16 ± 4.21b	72.27 ± 1.77a	51.07 ± 089b	0.7944 ± 0.21b	99.83 ± 0.04a
Freeze-drying	98.85 ± 0.90a	97.95 ± 4.21a	70.39 ± 6.20a	82.45 ± 0.89a	0.9350 ± 0.07b	99.84 ± 0.01a
Spray-drying	87.39 ± 0.45b	76.37 ± 7.37b	81.03 ± 7.08a	56.30 ± 3.55b	1.7844 ± 0.11a	99.65 ± 0.02b

Different letters in the same column mean significant difference at p ≤ 0.05.

**Table 6:** Glass transition temperatures of maltodextrin and encapsulated samples.

Products	Glass Transition Temperatures (°C)		
	Onset	Peak	End
Maltodextrin	153.98	155.34	159.62
Freeze-dried sample	146.34	148.25	153.72
Spray-dried sample	141.50	143.40	148.93
Microwave-dried sample	169.44	171.09	175.24

**Table 7:** Physical properties of microencapsulated powders.

Treatments	Dry Matter (%)	Water Activity	Bulk Density (g/cm^3^)	Tapped Bulk Density (g/cm^3^)	Carr’s Index (%)	Solubility (%)
Microwave-drying	98.23 ± 0.00a	0.0873 ± 0.0245b	0.49 ± 0.01a	0.59 ± 0.00a	17.00 ± 1.41c	99.95 ± 0.070a
Freeze-drying	98.10 ± 0.00a	0.0401 ± 0.0034c	0.11 ± 0.00b	0.15 ± 0.01c	27.00 ± 4.24b	90.45 ± 0.91b
Spray-drying	98.15 ± 0.00a	0.1627 ± 0.0004a	0.20 ± 0.00c	0.39 ± 0.01b	48.00 ± 0.00a	99.95 ± 0.070a

Different letters in the same column mean significant difference at p ≤ 0.05.

## Data Availability

Data sharing not applicable.
